# Better to Be Lucky Than Good: How Clinicians and Patients Frame Medical Outcomes

**DOI:** 10.1111/1467-9566.70276

**Published:** 2026-10-07

**Authors:** Eliza Brown, Stefan Timmermans

**Affiliations:** ^1^ Department of Sociology University of California, Berkeley Berkeley California USA; ^2^ Department of Sociology University of California, Los Angeles Los Angeles California USA

## Abstract

Notions of good or bad luck sit uncomfortably in contemporary medical practice defined by efforts to control life through biomedical knowledge and skilled expertise. Yet, talk about luck is common in healthcare to explain medical courses of action. Framing medical results as fortunate or unfortunate bridges the gap between predicted probabilities and variation in individual cases without undermining scientific neutrality. Luck mediates between the rational ideal of biomedical determinism that does not easily account for variable health outcomes and the magical invocation of faith or fate that denotes a moral judgement. Drawing from ethnographic studies of heart transplantation and fertility medicine, we find that luck is employed as an anticipatory frame to guide decision‐making in medicine and as a retroactive frame to justify unlikely outcomes. Invoking luck primes patients for the possibility of a favourable outcome and inserts a positive bias in medical care. The analysis underlines how luck frames in healthcare can shift attention away from structural reasons for disparate outcomes, attempt to absolve healthcare providers of blame from medical errors and promote continuing treatment. These findings indicate that focusing on when healthcare providers and patients invoke luck reveals the scope and limits of medical power and control.

## 1 Introduction

In a professional field organised around controlling life at the molecular and chemical level, luck is surprisingly omnipresent in medicine. Disease occurrence and biomedical treatments seem a luck of the draw: a non‐smoker dies from lung cancer in her thirties, whereas a neighbour who smokes a pack a day celebrates his 84th birthday. A baby undergoes newborn screening and the test results flag a rare metabolic condition, but the result turns out to be a false positive (N. Armstrong and Eborall 2012). Patients and care providers daily confront situations that defy biomedical logic but still happen. Good and bad luck may switch quickly across a patient's trajectory. Across healthcare settings, lucky coincidence may give way to unfortunate complications and what at first seems like an unlucky series of events may have a distinct silver lining.

Luck has recently come to the attention of scholars as indispensable for understanding stratification and life course outcomes (van de Rijt et al. 2026; Rank 2024; Streib 2023). Sauder (2020) has argued that sociologists should take luck more seriously because ignoring the role of chance misrepresents the social world as overly rational, structural and meritocratic. Sauder defines luck as involving chance, consequential (either beneficial or harmful) and outside the control of the people affected (Sauder 2020). Sauder encourages studying social phenomena ascribed to luck as opposed to other explanations (see Loveday 2018).

Following Sauder, we examine luck as an interpretive frame that patients and healthcare providers rely on to make sense of a medical course of action. Unlike Sauder, we view luck not as universally valued (even when most agree that enjoying good health is luckier than poor health), but rather as something that is socially produced through interaction, particularly in medical contexts defined by asymmetric expertise. We emphasise that a luck framing is in constant tension with efforts to increase predictability and control over a medical course of events. When people ascribe medical events to luck, they circumscribe biomedical acumen and patient agency. The framing of biomedical events as fortunate or unfortunate not only stands in tension with biomedical explanations of control, but also with alternative frames such as fate and faith.

We draw from two research projects where the element of luck is widely recognised: heart transplantation and fertility medicine. With thousands of patients on the waiting list, organ transplantation rests on an organ becoming available at the right time. Fertility medicine depends on improving the chance of pregnancy over nonmedicalised conception, but fertility providers cannot guarantee success. In both medical areas, health professionals aim to exert control over an inherently uncontrollable process, generating opportunities for framing outcomes as luck.

Our contribution is twofold. First, we show that medical providers and patients regularly invoke a luck frame. As much as medicine strives for biomedical control, medical providers and patients alike reach for luck as one frame to bridge the gap between probable and actual results. Ritual, faith and magic have long been identified by sociologists and anthropologists as part of medicine—from Evans‐Pritchard's (1937) study of Azande witch doctors to Hughes' (1971 (1945)) writing of medical providers' rituals as bulwarks against the risks of the trade—but luck has gone undertheorized in the literature even as it has paradoxically remained prominent in medicine following the epidemiological turn. Risk assessments and increased awareness of what could and should transpire based on probabilities allows for luck to mediate between plans and outcomes using statistical language (Aronowitz 2008; Kolker 2004).

Second, we argue that, unlike discourses that are only anticipatory—such as faith—or only retrospective—such as fate—luck is employed both as an anticipatory frame to guide decision‐making in medicine and as a retroactive frame to justify outcomes. Providers explain to patients which outcomes are most likely, along with those that they might hope for with a little luck. Conversely, medical outcomes might be attributed to disease pathology, human intervention or God's plan, but they also might be attributed to luck—particularly in cases marked by poor outcomes that cannot be easily explained or would have unfortunate complications. This dual anticipatory and retrospective quality contributes to the utility of a luck frame in many medical situations. We argue that resorting to a frame of luck reinforces biomedical dogma by instilling hope that a fortunate outcome remains possible.

## 2 Framing Medicine

How people make sense of situations shapes their plans for future actions, the judgement of past actions and the motives they impute to others. They do so by employing frames (Goffman 1974) or schema (Schutz 1967), or what we refer to as interpretive frames. Medicalisation may be considered a meta‐frame that competes with alternative frames such as criminalisation (Conrad and Schneider 1980). Within medicine, medical sociologists have attended to the interpretive frames invoked by clinicians and patients to understand medical events, diseases and patient populations (Aronowitz 2008). For example, Martin (1987) showed how framing eggs as passive and sperm as active in medical textbooks influenced research in scientific labs. Interpretive frames do something: how people understand a situation prompts social action, it privileges one reality over possible other ways of interpreting a situation (Benford and Snow 2000; Hacking 1999). We are particularly interested in luck as a frame to explain why medical interventions might succeed or fail and what framing an (anticipated) outcome as fortunate or unfortunate does for the limits of medical acumen and patient responsibility. The distinctive reality luck highlights makes sense through its relationship with other common frames used to explain the health interventions, including biomedical essentialism, medical skill, faith and fate.

First, medical professionals might turn to *biomedical explanations* grounded in the evolving medical knowledge base. This interpretive frame suggests that increasing medical control is fundamental to the biomedical project. This framework rationalised by scientific principles, bureaucratic protocols and biotechnologies, expunges mystery and enchantment from medicine (Weber [1930] 2002). Within a biomedical frame, disease‐complication is expected and explainable by genetic mutations, poor health behaviours, infection, graft rejection, social determinants of health, among other causes. The medical profession sets itself apart through its control of biomedical knowledge and use of clinical language (Freidson 1970; Gieryn 1983; Anspach 1993). We might expect this to be the dominant frame for explaining medical phenomena among medical professionals generally, as well as patients with a high degree of trust in the medical establishment.

Second, another ready explanation centres on *medical skill.* Different from the biomedical model that suggests a cognitive front, with this interpretive frame beneficial medical outcomes might be attributed to clinical expertise and poor outcomes can be explained by shoddy medical work. Indeed, there is a wealth of medical sociology on clinician error, such as medical trainees learning from mistakes (Bosk 1979; Brewer et al. 2020). Beyond the skill of the individual clinician, medical organisations might be marked by inadequacy or competence and patients and providers might point to organizational dynamics and reputations to understand outcomes (Brown 2023). On the patient side, healthism (Crawford 1980) also relies on an interpretive frame of responsibility and skill to understand medicine: a patient does not want to be operated on by any clinician but by the best specialist in the field. The framing of medical outcomes resulting from technical skill is related to the merit frame for justifying educational or professional success (Friedman et al. 2024; Sauder et al. 2024). This interpretive frame particularly resonates with elite clinicians working in high status organisations (Jenkins 2020) or with patients who believe that they are better at maintaining their own health than most of the population.

Third, patients might turn to explanations that rely on *faith, karma* or *fate* to understand medical processes, reintroducing mystery, enchantment and magic. This interpretive frame abdicates control over outcomes to a higher power and assumes that outcomes are predetermined by factors out of human control, often leading to a fatalistic attitude. Religious patients might consider themselves ‘blessed’ when medical interventions go well (Long et al. 2024). Still, fatalism is not a personality trait opposed to individual agency that exists across contexts (Drew and Schoenberg 2011; Bell and Hetterly 2014): structural and cultural factors might prompt some patients to ‘choose’ fatalism when other medical choices are arduous or impossible. We might expect this interpretive frame to be resisted or sidestepped by clinicians habituated to understanding outcomes as the result of biomedical knowledge or technical skills, although they too might point to destiny when outcomes occur that they believe were beyond their ability to control (Roberts 2012).

These frames are not mutually exclusive but often co‐exist. Fox (1957), for instance, showed that one key challenge facing medical students learning how to doctor was to figure out whether uncertainty in medical practice was due to gaps in the medical knowledge base or in their own technical skills. Religious patients may acknowledge their medical team's role in restoring their health while also believing that God answered their prayers, generating a stronger locus of internal control (Koenig 2012).

Why, then, do people turn to luck as an interpretive frame in medical situations? Luck carves out a different space from biomedical determinism, skill, fate or faith in that luck does not claim total human control nor does it abdicate control to a higher power. Luck is one way to mediate between rational and magical logics (Parsons 1951). And unlike other frames such as religious discourse (Roberts 2012), by using the language of probability, luck does not chafe against scientific sensibilities.

Luck is an explanation that can help resolve clinical uncertainty or ambiguity. Although uncertainty can sometimes be a useful tool for medical providers to motivate patient behaviour (Timmermans et al. 2018), providers still regularly attempt to resolve diagnostic uncertainty by reaching for other explanations (Brown 2020). Luck can prove particularly useful for understanding the meanings and weighing the merits of multiple options depending on their likelihood and valence. In the context of explaining occurrences, luck differentiates the outcome a patient is experiencing from the many that could have happened. In the context of decision‐making, luck implies that making decisions renders certain outcomes possible with some more likely than others, without determining which outcome will prevail. The future remains hazy but luck influences which of the possible outcomes medicine obtains. It may apply to situations where the intervention was executed perfectly but the outcome was not as expected (e.g., the surgery was a success, but the patient died) or when prayers remain unanswered. Fate and luck differ in the sense that fate requires that actors *stop* attempting to change their futures because outcomes are predetermined (e.g., it was fated), luck does the opposite by prompting social action to improve future changes (e.g., making one's luck). In which kind of medical situations is luck then invoked? Anything could be lucky or unlucky, but patterning tells us about how healthcare providers and patients see medicine's powers to control.

## 3 Methods

This article draws from two independent studies: heart transplantation and fertility medicine. We selected these cases because luck is a particularly salient interpretive frame in both, although in different ways, as described below.

The fertility medicine case comes from ethnographic fieldwork conducted at three fertility clinics in the United States in 2018–2019 which were selected to represent different organizational structures and medical philosophies in the fertility field. This fieldwork focused on patient consultations (272 total), while also including other clinical activities such as grand rounds, staff meetings and support groups. Patients consented to participate in a study about people's experiences undergoing fertility treatment before consultations at the clinic. As such, these findings are especially focused on the role that luck plays in planning for future treatment, which often includes adjudicating why past treatment failed. Brown took notes directly during consultations as well as immediately after and wrote memos throughout her ethnographic observations on recurring themes. Brown specifically coded for conversations around ‘chance’ and ‘luck’ in fieldnotes. There were important differences between the fertility clinics regarding organisation and ownership, clinical approaches and philosophies, size and targeted clientele, among other features, but at all clinics, consultations included significant conversation around luck. This study was approved by New York University IRB. Readers can find more details about the clinics and research methods in (Brown 2025).

The heart transplant case comes from ethnographic fieldwork conducted at one of the US leading heart transplant centres during 2024–2026. This fieldwork followed patients through the process of transplantation from the moment a heart transplant was raised as a possible treatment, through the selection process, the waiting time on the transplant list, the transplant and 1‐year transplant experience. The research consisted of following interdisciplinary transplant teams on clinical rounds in the hospital and in cardiac clinics, attending selection committee meetings and interviews with patients and care providers. Timmermans followed 51 patients through this process. Patients voluntarily consented to participate in a study on the role of social support in heart transplantation. The theme of luck emerged as a central issue based on the number of elements that need to align to make it into a transplant programme and occurred throughout the transplant process as a recurring explanatory framework for why patients did well or not. The findings focus on the role luck plays in getting listed for a transplant, the time to get a transplant and the outcomes post‐transplant. This study was approved by University of California, Los Angeles IRB. Additional methodological details about this study can be found in (Timmermans, under review).

We individually coded our own field notes and independently identified luck as a major sociological theme. Subsequently, we discussed coded excerpts together to determine how luck discourse was employed by medical providers and patients and wrote notes from these conversations. Through this iterative coding process, we abductively developed a theory of luck in medical situations informed by the sociological literature on luck, looking for surprizing findings in light of the literature (Timmermans and Tavory 2022). As part of this process, we also discussed negative cases, such as when medical providers argued that no amount of luck might lead to a desired outcome, which ultimately strengthened our theory around luck mediating between possible outcomes but not allowing for eventualities deemed impossible.

Although in the fertility project, luck was often invoked to anticipate possible outcomes, and in the heart transplant project, luck often justified waiting time and transplant outcomes, in both projects, we noticed that luck put a positive spin on the medical process, giving patients hope that a favourable outcome was possible. These findings directed us to the sociological literature on framing, which forms the theoretical foundation for this article. We analysed the data for variation in the use of luck framings, taking advantage of the different perspectives (patient and healthcare provider) and the different research sites. The commonalities rather than the differences stood out between the two research projects.

## 4 Luck in Accessing Medical Care

Even before a patient sees a clinician, health professionals and patients may recognise that luck played a role at different levels of the healthcare system. Sometimes this is in the broad sense of being born at this moment in history in which certain medical technologies are available. For example, one fertility specialist reassured a distraught couple undergoing in vitro fertilization (IVF) that although they were unlucky to be experiencing infertility they were lucky to do so today: ‘here's technology that is available to help that wasn't available 30, 40 years ago and thank God it's here, there are a lot of people that we couldn't help before’.

Clinicians may point out that a patient must be the right person at the right time at the right place to activate medical care. In heart transplant medicine, clinicians examine not only whether a patient is sick enough to qualify for a transplant but also whether they are well enough to tolerate such an invasive surgery. Unfortunately, some patients who were asked about a transfer to a transplant programme decided to wait because they felt well. By the time they were ready for a transplant, their condition had weakened to the point that transplant surgery was no longer an option. In such a situation, an attending cardiologist told a resident: ‘I wish we had seen him 6 months earlier’. Alternatively, some patients get a lucky break. In the transplant project, a patient's wife advocated strongly for her husband on life support to try anything to save him when the ICU staff at the regional hospital initiated conversations about withdrawing care and letting the patient die. The ICU staff was receptive and contacted several heart transplant programs in the region. One programme happened to have a free bed and the patient was transferred and eventually received a transplant. These patients stand out as particularly unfortunate (accessing rare lifesaving care but not being eligible) or lucky (accessing rare lifesaving care in the shadow of death) in a context that is structurally stacked against some populations accessing care.

Although reflecting on the alignment of factors required to access medical care, healthcare professionals acknowledge that there is a large place for luck in the medical enterprise. Such statements reassure patients that accessing medical care is already a fortunate outcome. They could delineate external from internal medical care framing: outside you had to be lucky to interpret your symptoms correctly and seek medical help, but once inside, we have it under control. That is not the message that patients receive. By receiving healthcare from the current clinician, patients have a chance at a positive outcome, but that outcome still requires luck. For example, in a fertility consultation, a specialist explained to a patient that you cannot control getting a ‘good’ embryo (i.e., one with 46 chromosomes), ‘it's dumb luck, statistics and odds’. Here, the specialist qualifies the luck involved in the genetic composition of embryos as ‘dumb luck’, as opposed to something that might be enhanced with medical skill or patient compliance. In the heart transplant case, a patient waiting for a donor heart was told ‘it can be a day, it can be a week. We don't have any control over it’, implying that the patient will need a measure of luck. Clinicians use luck to signal to patients the boundaries of their control. They typically refer to luck prospectively––to set expectations––and retroactively––to explain fortunate or unfortunate outcomes. They also use luck to reframe potential bad situations by pointing out a silver lining. We will turn to the use of these luck framings next.

### 4.1 Framing Luck

#### 4.1.1 Anticipation

Because clinicians cannot guarantee outcomes, luck is a necessary ingredient for things to work out in medical practice. Clinicians lay out for patients what is likely to occur based on existing evidence along with what they hope might occur. Although it might seem as though the utility of luck might decrease with the turn towards evidence‐based medicine, in fact, luck is especially useful when making decisions using population‐based models in clinical work (D. Armstrong 2002). It explains the difference between what should be occurring on a population level to what is happening on an individual level. Even when patterns are clear, it is not obvious how they might manifest in a particular person. This unpredictability necessitates luck. To take an example from the fertility study, a fertility specialist explained the odds of preimplantation genetic testing for aneuploidy (PGT‐A) to a patient, a step in which an embryo is biopsied and then those cells are tested for an abnormal number of chromosomes, as reflected in fieldnotes:

At the patient’s age, only about 10% of embryos are normal, so she would need to get 10 embryos to get one normal embryo, adding that she has to get a little lucky and people get lucky around here every day.

There are population‐level calculations, but these estimates do not definitively predict what will happen in her case. There needs to be some transformation from numerical probabilities into personal meaning (Adelsward and Sachs 1996). Luck mediates this disjuncture: Luck would allow her to perform better than expected for her age.

In this way, luck works within expected parameters of evidence‐based medicine. Like the medical aphorism, ‘When you hear hoofbeats, think horses, not zebras’, luck can bridge the gap between the expected results and the hoped for results, but it cannot break known scientific laws. Clinicians are not expecting unicorns. To continue with the same fertility case, the physician explained to the patient that she might not have 10 embryos for them to test, but she might have three or more. He expounded that:

sometimes people who only have three embryos to test get lucky and one out of the three is normal, but that some people have eleven embryos to test and they all come back abnormal—it just happens.

In this case and many others like it, luck works as a frame for understanding the range of potential outcomes. As with outcomes, luck can work in either direction around expectations. Sometimes the expectation is for swift recovery or a good response to treatment, but bad luck may keep you from the desired outcome. Other times the expectation is that the odds are against you, but you may get lucky. Luck then works as a final component of medical practice that can make the patient defy expectations, for better or for worse.

As an anticipatory frame, clinicians mostly invoke luck to prepare patients for a positive outcome. In looking to the future, hope provides the ‘emotional substratum’ (Mische 2009) between the reality of what is and the anticipation of what could be. In this sense luck as an explanation and hope as an emotion are distinct but often entangled. Patients might hope for good luck prior to undergoing medical procedures or surgeries. For physicians as much as patients, luck allows for instilling hope to ‘beat the odds’. To plan to undergo the arduous IVF process, the physician and the patient need to consider the most likely path of attrition, but also the path in which they may get lucky. In the heart transplant case, patients could be unlikely to receive a heart transplant because of their height, weight and blood type—but it would be counterproductive to just point to their low odds of getting a transplant and stop caring for them. Instead, transplant cardiologists remind patients that ‘you never know’ and a ‘perfect’ heart may become available.

Luck is not only used to instill hope but also to temper expectations. In the fertility medicine case, physicians are careful to avoid promising specific outcomes from treatments. Even for the best prognosis patients, the success rate for an IVF cycle is just over 50%. Patients and providers alike need luck to be on the right side of that bet. This came through in patient‐provider communication about treatment decisions. For example, in one fertility consultation, a patient asked her physician if he typically sees success with patients on their first round of IVF or if it takes more than one round. The physician responded, ‘hopefully it takes only one but there's never a promise that it takes only one’. After repeating her chances of getting pregnant from one IVF cycle discussed earlier in the consultation (around 25%), the physician added, ‘we need a little luck’. In this case and many others like it, the patient certainly could get pregnant from one round of IVF—if they are lucky—but she just as easily (in fact, more easily from a statistical perspective) could not. Similarly in transplant medicine, a cardiologist told a patient: ‘We know that the odds of living longer are higher with a heart transplant than doing nothing. But there are no guarantees’. Hope and tempering of expectations occur in the same exchange, underscoring the dual message of resorting to luck talk: good things are possible, but the health professionals cannot guarantee them. In the meantime, they encourage the patient, as a transplant cardiologist put it, ‘to hang in with them’. Pointing to luck then encourages patients to stick with the medical programme because even if there are no guarantees the potential upside makes it worthwhile.

#### 4.1.2 Justification

Providers and patients may also invoke luck after medical events, particularly when ascribing or diffusing blame to medical failures. Risk might describe probabilistic chance, but it is also a moral judgement about responsibility (Douglas 2013). Medical providers might point to ‘bad luck’ rather than medical error, shoddy work or personal failing as a reason for undesirable outcomes. In this way, framing bad outcomes as bad luck is a recurrent interactional approach with the aim of absolving medical providers and systems of responsibility (and potential litigation). It also can potentially absolve patients from personal health responsibility or the guilt that they could have done more to improve their health outcomes based on their lifestyle or compliance. For these reasons, justifying results through a luck framing could be contentious, in line with prior work on contesting accidents (Green 1997).

In the fertility case, treatments not resulting in pregnancy were often ascribed to bad luck. But whether the outcome should be blamed on bad luck, the wrong approach or a mistake had to be negotiated in medical encounters. For example, a patient switched from a boutique clinic to a budget clinic to save money, with the idea that she just needed to do more IVF cycles anywhere and eventually she would get lucky. Her doctor at the boutique clinic said as much saying, ‘you're rolling a dice [sic]. With a 10% chance, you have to roll the dice 10 times’, suggesting that her poor results were simply a matter of chance. After failing to get pregnant at the more affordable clinic, she returned to the more specialised and expensive treatment, saying that she thought that was what she was doing—rolling the dice a few more time—‘but nothing is that easy’. Instead of attributing her poor outcomes to luck, she believed that her poor outcomes were the result of a lack of skill on the part of the budget clinic.

In the heart transplant case, the negotiation of bad luck may still turn into a discussion of medical neglect and error. Soon after a patient received a transplant, the team discovered that the donor had cancer. They discovered some small lesions in the heart and consulted an oncologist, who reassured the team that it was a negligible finding. The patient was soon diagnosed with rapidly metastasising cancer and his wife and daughter resisted every notion of this outcome being due to bad luck. They insisted that medical malpractice was the reason the patient was now dying.

Yet, even if luck might be employed to absolve medical providers of responsibility for poor outcomes, sometimes the assessment of ‘bad luck’ can propel patients and providers into further medical action by renewing hope. In the fertility case, with a good‐prognosis patient who had undergone three textbook embryo transfers without getting pregnant, the fertility specialist that she met with suggested that it was possible that the patient just had had bad luck three times rather than that something was wrong with her embryos or attributing the failures to lack of medical skill. Rather than absolving responsibility, this assessment allows the patient and provider to consider further treatment as potentially successful, rather than doomed before even attempting. In this case and others like it, following procedures is the best warrant for ascribing poor outcomes to bad luck rather than to mistakes (Bosk 1979). It is this sense that even if one follows all protocols that luck remains a necessary ingredient that led one fertility specialist to surmise, ‘better to be lucky than good’.

#### 4.1.3 Adding a Positive Valence to Chance and Uncertainty

Both prospectively to prepare patients for the range of outcomes and retroactively to explain the medical course that enfolded, providers and patients invoked luck to add a positive valence to chance and uncertainty. Although theoretically clinicians could engage in luck talk to cast a pessimistic cloud over the medical events, medicine trends towards therapeutic optimism (Jaffe et al. 2021) and clinicians invoke luck to put a motivational spin on the events. Every patient is almost by definition unlucky if they need medical help, but providers and patients alike might talk about ‘silver linings’ of being struck with the bad luck of needing medical attention (Stivers and Timmermans 2017). Through the framing ‘it could have been worse’, good luck more than bad luck becomes the dominant narrative.

One ‘silver lining’ might be a greater appreciation for life's gifts. In the fertility case, one fertility specialist told a patient that when she has kids, those kids would be the ‘luckiest kids in the world’ because the patient understands what a gift and privilege it is to have kids. The physician went on to say that he has been working in fertility medicine for years and he has seen the kids resulting from fertility treatment do amazing things. ‘Why?’ he asked rhetorically, ‘because of their parents who worried that they would never have a baby and then appreciate their children. It's a little silver lining. You learn a lot about life and about yourself’. This fertility specialist reframed the bad luck of infertility with the good luck of having good children, suggesting struggle yielded the ‘silver lining’ of appreciation. In the transplant study, a stand‐up comic who had undergone a heart transplant and was asked to talk to an annual transplant celebration, greeted the audience with: ‘I have been on many stages in my life, but I don't think I have ever been in front of a group of the luckiest people on earth’.

An optimistic bias aims to keep patients enroled in medical care. Patients often push away bad prognoses. As bad as the outlook might be, there is the sense that they could still get lucky. This tendency towards optimism is perhaps why people rushed to use IVF when it was first invented and had very low odds of resulting in children (Franklin 1997). But it is not only patients who tend towards optimism. If patients hesitate about continuing treatment, clinicians highlight how other patients were lucky to stick with the programme. Patients who might want to quit treatments are persuaded that there is another treatment they can try. During a fertility clinic support group meeting, a patient's husband asked the nurse facilitating the group if there is ever a point where it's time to stop. The nurse responded, ‘we don't tell you that’. She said that they will give them other options, concluding: ‘There is always something we can offer’.

### 4.2 Making Your Own Luck: Coupling Luck With Other Explanatory Frames

#### 4.2.1 Coupling Luck and Biomedical Knowledge and Skill Frames

Although clinicians readily admit that a patient requires some luck and that fluke events happen with fortunate or devastating outcomes, they also aim to curtail the role of luck in patient care. A transplant cardiologist has little control over when a donor organ becomes available and some patients are lucky with a short waiting time, but they aim to optimise the patient's mental and physical condition for when the transplant happens. Because listing for transplant depends on a patient's priority based on UNOS criteria, clinicians may upgrade a patient's status on the waiting list by adding mechanical support devices.

Looking at a patient’s chart and noting deteriorating cardiac and renal function compared to yesterday, Dr. Nedialkov observed: ‘This guy is a ticking time bomb’. After some reflection, she turns to the cardiology medical student and says, ‘let’s put him on a balloon pump so we can make him status 2.’

The cardiologist decides to compliment the patient's pharmaceutical cardiac support with an intra‐aortic balloon pump, a device to increase cardiac output. This changes the patient's UNOS priority from a status 3 to a status 2, allowing for a higher priority listing (a lower number means a higher priority) and, hopefully, a quicker transplantation time. It is also a gamble. The patient's mobility will be limited with a balloon pump, which may lead to infections and physical decompensation resulting in a more challenging post‐transplant recovery.

Much of medical care, both preventative and curative, can be understood as tipping the scales towards positive outcomes while controlling as much of the clinical situation as possible. Relying solely on luck diminishes professional expertise because it suggests that medical intervention is a mere matter of chance. Instead, lucky or unlucky circumstances nudge clinicians to learn about what went right and wrong and setting patients up for success. The claim to professional expertise depends on doing better than chance. The luck and medical knowledge‐expertise frames blur in these situations. The role of luck is acknowledged but some clinical interventions may tip luck in a more favourable direction.

Several institutional mechanisms exist to probe bad luck or carry forward the lessons from fortunate circumstances. Much of informal teaching during hospital rounds consists of senior clinicians sharing their experiences about clinical symptoms that are ignorable or other ones that at first sight are not alarming but may cascade into devastating outcomes. When patients develop complications, these same mentors put junior physicians on the spot by asking them why, for instance, the platelet count is low or creatinine levels do not budge after diuresis. If patients die, the mortality and morbidity conference provides a deep dive in the medical file combined with findings of the pathology report to separate luck from understandable pathological processes. A lung tumour hiding undetected behind an implantable defibrillator is not just bad luck for the patient but also a teaching point to imprint on future generations of clinicians. In this way, luck is channelled into gaining control through medical knowledge.

Medical practice and outcomes are contingent or dependent on conditions being met as well as chance events (Pope 2002). Based on iterative learning curves drawn from accumulated experience, clinicians aim to up the odds for positive outcomes. At the transplant selection committee meeting, a surgeon remarks ‘He is a big O, maybe we should consider an LVAD (Left Ventricular Assistive Device, an implantable heart pump)’. This suggestion aims to tip the luck balance for a particular kind of patient. The waiting time for an organ depends on blood type, patient size and weight (also antibody matching). O positive blood types are universal donors. A patient needing a transplant with an O positive blood type may wait longer for a donor heart to become available because any patient with another blood type who is ranked a higher priority may receive the first available O positive heart. The O positive patient, unfortunately, cannot receive an A, B, AB or O negative blood type heart and those hearts may go to lower priority patients matching that blood type. This disadvantage is compounded for large patients because they can also not receive an undersized heart and more heart donors have small bodies. Small patients, however, can accept slightly larger hearts. The surgeon's suggestion aims to stave off the unfortunate situation where a large, O positive patient deteriorates on the waiting list and is no longer eligible for a heart transplant. Large, blood type O patients in precarious condition may therefore be redirected to a durable support device, an LVAD, as a bridge to transplant. After receiving the LVAD and a healing period of 6 months, these patients can be reactivated on the heart transplant waiting list. The LVAD gives them a high priority listing and can keep them perfused several years while waiting for a donor heart. Here we can see where medical skill and luck meet: providers cannot necessarily make any outcome happen for any patient, but they can learn how to improve their chances through clinical expertise and biomedical knowledge.

In fertility medicine, switching clinics often works as a way of making new luck by seeking out a different treatment approach more likely to result in pregnancy (see Brown 2023). For example, at one clinic, a fertility specialist explained to a patient what he might do differently compared to her previous doctor but added that he could not tell her that she absolutely needed these additional treatments because ‘it could still be a matter or chance and not being lucky’. Still, he explained that if she was asking about the extra things that ‘one might do to increase her chances’ then these would be his suggestions. Pure luck means that switching clinics or approaches does not matter—making luck by increasing one's chances, in contrast, suggests that one can optimise by finding higher quality or better matched care.

#### 4.2.2 Coupling Luck and Fate‐Faith Frames

If we think of medical framings as a continuum of human control with medical knowledge on one side and fate‐faith at the other with luck in the middle, health professionals may also influence a fortunate outcome by courting fate. Across the studies (and in the literature [Bosk 1979]), clinicians set up patients and themselves for good fortune by incantating ‘knock on wood’ or ‘fingers crossed’.

A physician looking over the ward’s census absentmindedly observed that ‘We don’t have people on the ICU anymore?’ With dramatic astonishment, her supervisor turned to the team and said: ‘Did she just say that out loud?’ The physician, realizing her mistake, ran to the closest door and knocked on it while laughing.

By expressing that the census was low, the clinician may have jinxed the team and brought on an onslaught of new patients, bad luck for everyone involved. Knocking on wood reversed this spell.

Clinicians engaged in these rituals not only to ward off heavy workloads, but also to assure successful surgical outcomes. When a heart became available for donation, the cardiologist would inevitably warn the potential recipient that the surgeons may decide that the heart was unsuitable. This decision may even be made after the patient was anesthetised in the operating suite. A cardiologist would admonish a patient that they only knew for sure that they received a heart transplant if they woke up with one, adding, ‘fingers crossed’ while showing crossed fingers on both hands. These superstitious gestures aimed to secure a tincture of extra luck, biomedical faith against fate, to prevent adverse outcomes over which the team had little control.

In the fertility case, luck sometimes mixed with faith, especially during the time between treatment and evident results (see Roberts 2012 as another example). Rituals were critical at embryo transfer or insemination to ensure luck prior to entering into this period in which possible impregnation is black boxed inside the body, colloquially referred to as ‘the 2 week wait’. Medical superstitions such as having patients lie down with their feet up for an extended time after an embryo transfer abounded and patients took part in rituals propagated in Internet chat rooms such as eating McDonald's fries. At one of the clinics, a few of the physicians prayed with patients after transfers. For example, at the end of one embryo transfer, the physician held hands with the couple and told them, ‘not to go chasing pregnancy tests’. He then asked if they wanted to say a prayer, to which they nodded. He said a prayer for pregnancy addressed to Jesus Christ and they all said amen. Patients and providers hoped rituals like this one would improve the luck of these medical interventions, not displace them.

In these situations, the line between luck and fate‐faith framing blurs. Making your own luck remains intangible and taming the intangibility by producing something that is intrinsically out of human control, becomes an invitation to magical and religious thinking. Healthcare providers and patients may thus acknowledge the importance of luck in getting a new heart or a viable embryo but still resort to superstition or prayer to tip luck in a favourable direction. Superstition and prayer are accepted in these situations because even if wearing lucky socks, whispering a prayer or crossing fingers may not help in summoning luck, it will not hurt either.

## 5 Discussion

Clinicians thus have a mixed attitude to luck: they realise that luck plays a dominant role in clinical care, but they do not want to depend on it, even if they want to set their patients up for good fortune. This mixed attitude towards luck has several consequences. First, it can obfuscate the ways that biases persist in the healthcare system by ascribing them to luck. The heart transplant field, for instance, has geographical disparities in access to programs, with some states lacking programs and other states excluding Medicaid populations from heart transplants (Daw 2015). Thus, framing a heart transplant as a lucky outcome for a patient with advanced heart failure ignores the uneven geographical distribution of heart transplant programs in the country, glosses over the persistent racial and gender inequities of heart transplant receipt and survival and avoids confronting that someone had to die with still usable organs for a different patient to live. In the fertility case, physicians framed the technological development of IVF as lucky to patients, a silver lining of experiencing infertility, while eliding the fact that most people who experience infertility in the United States of America do not have access to IVF because of its expense and the limited number of clinics outside of major cities (Tierney et al. 2024). In that sense, luck often works as an explanation that leaves unexamined an unequal health system.

Second and more generally, while an unfortunate medical outcome may be probed for factors that are under medical control, ascribing the outcome to bad luck may also close the opportunity for further reflection. Mistakes may be viewed as unfortunate and therefore inevitable events or they may lead to a review of a sequence of events that led to an unfavourable outcome. The organizational issues that can go wrong in medical practice are endless. Within this institutional landscape, some patients will be exceptionally lucky, whereas others experience delays and complications. Whether a patient is lucky or not, however, depends not only on what happened but also on whether what happened is recognised as such.

Third, counting or even hoping for luck may also skew medical decision making when clinicians make decisions based on anecdotal rather than evidence‐based knowledge. Clinicians share stories of patients who have beaten the odds or single case reports of exceptional patients. Although a single refutation can destroy a mathematical proof, in medicine exceptions are common. The problem occurs when a single lucky outcome sets clinical practice resulting in availability bias. Anecdotalism refers to believing in the exception rather than the rule. Luck may not be enough, sometimes a patient will not just need luck but a ‘miracle’ to pull through and clinicians opt for a ‘Hail Mary pass’. This often involves situations where clinicians get emotionally attached to a patient or the patient is exceptionally motivated and no one wants to tell them that they have reached the limit of medical care.

One of the key characteristics of luck is its utility as a framework both in anticipating outcomes as well as justifying outcomes. In this sense, providers can seamlessly set up for patients that they are operating in the realm of chance and then describe what transpired without shifting frames. Because in medical situations luck is so often socially produced under conditions of asymmetric expertise, this continuity is important for maintaining medical trust and authority. Patients might be more likely to take up luck frames that have been applied consistently, as opposed to luck frames that seem to be taken up suddenly to avoid the appearance of error or injustice.

Moreover, comparing the value of luck as an explanation is made plain through juxtaposition with situations in which magic, religion and uncertainty dominate as terms. Indeed, in cosmetic surgery, medical providers' transformative work as evidenced by striking ‘Before and Afters’ might be discussed as performing magic or witchcraft (Menon 2023); in end‐of‐life conversations, providers and patients alike tend towards religious discourse (e.g., Seale 1995); and in emerging medical fields like genomics, practitioners might reach for metaphors that convey uncertainty (Timmermans and Buchbinder 2010). A luck framework does not crowd out these other frames but has its own place in the explanatory realm. Illness and disease provoke existential questions: How do you explain why some die while others live? Luck is not always up to the explanatory task, but in many medical cases—particularly those that emphasise population statistics and clinic averages—practitioners and providers tend to point to luck as a way of understanding the world without casting blame or taking responsibility.

## 6 Conclusion

Framing medicine as luck thus does something specific in medicine. Clinicians and patients use luck to anticipate outcomes from medical interventions in situations where they have little control over the result. They also deploy luck to understand and justify beneficial or harmful outcomes of interventions after the fact. Rather than increased rationalisation diminishing the role of luck in medicine, luck framings enhance it through articulating what could have happened. Attending to the different instantiations of luck as an interpretive frame, we can juxtapose understandings of biomedical and technological determinism, medical skill and deservingness and medical fatalism. We can understand what medical providers and patients perceive as not under their control by observing what medical providers attribute to luck. When a fertility doctor tells a patient that the number of genetically normal embryos is a matter of luck, she is saying that she can control something—the production of embryos outside the body via IVF—but not everything. When a heart transplant surgeon attributes a failed transplant due to infection to the patient's neighbourhood fungal biome, she is saying that she can control most of what happens within the hospital walls, even if she loses her grip on what transpires outside. Luck frames both the object and a negative space. In that sense, luck protects biomedical knowledge and medical expertise framings while minimising the role for faith‐fate framings. Luck mediates between full medical control while acknowledging that some control is possible and predetermined destiny while realising that some aspects are indeed out of medical control.

Luck changes medical practice; it gives people hope. The fact that medicine cannot control it all makes outcomes not deterministic. Even if there is a 95% chance of bad outcomes, maybe you will end up in the 5% who beats the odds. And if not this time, maybe next time. Good luck will prevail. Medical interventions like CPR that rarely work still might work this time—but if you do not try, you do not know (Timmermans 1999). In this way, luck framing reinforces the biomedical project: If you stick with medicine, you too could get better outcomes. Medicine, then, seeks to constantly narrow what is left up to luck and frame outcomes as the result of improved science, but at the same time depends on luck to promote itself and keep hope alive, for both patients and providers.

## Author Contributions

**Eliza Brown:** conceptualization, writing – original draft, methodology, writing – review and editing. **Stefan Timmermans:** conceptualization, writing – original draft, writing – review and editing, methodology.

## Conflicts of Interest

The authors declare no conflicts of interest.

## Data Availability

Research data are not shared.
